# Identification of Emotion Using Electroencephalogram by Tunable Q-Factor Wavelet Transform and Binary Gray Wolf Optimization

**DOI:** 10.3389/fncom.2021.732763

**Published:** 2021-09-08

**Authors:** Siyu Li, Xiaotong Lyu, Lei Zhao, Zhuangfei Chen, Anmin Gong, Yunfa Fu

**Affiliations:** ^1^School of Information Engineering and Automation, Kunming University of Science and Technology, Kunming, China; ^2^Brain Cognition and Brain-Computer Intelligence Integration Group, Kunming University of Science and Technology, Kunming, China; ^3^Faculty of Science, Kunming University of Science and Technology, Kunming, China; ^4^School of Medicine, Center for Brain Science and Visual Cognition, Kunming University of Science and Technology, Kunming, China; ^5^College of Information Engineering, Engineering University of PAP, Xi’an, China; ^6^Computer Technology Application Key Lab of Yunnan Province, Kunming University of Science and Technology, Kunming, China

**Keywords:** emotion recognition, emotional brain-computer interface, tunable-Q wavelet transform, binary gray wolf optimization algorithm, EEG

## Abstract

Emotional brain-computer interface based on electroencephalogram (EEG) is a hot issue in the field of human-computer interaction, and is also an important part of the field of emotional computing. Among them, the recognition of EEG induced by emotion is a key problem. Firstly, the preprocessed EEG is decomposed by tunable-Q wavelet transform. Secondly, the sample entropy, second-order differential mean, normalized second-order differential mean, and Hjorth parameter (mobility and complexity) of each sub-band are extracted. Then, the binary gray wolf optimization algorithm is used to optimize the feature matrix. Finally, support vector machine is used to train the classifier. The five types of emotion signal samples of 32 subjects in the database for emotion analysis using physiological signal dataset is identified by the proposed algorithm. After 6-fold cross-validation, the maximum recognition accuracy is 90.48%, the sensitivity is 70.25%, the specificity is 82.01%, and the Kappa coefficient is 0.603. The results show that the proposed method has good performance indicators in the recognition of multiple types of EEG emotion signals, and has a better performance improvement compared with the traditional methods.

## Introduction

Emotion is a psychological phenomenon mediated by the subject’s needs and desires. It has three components: physiological arousal, subjective experience, and external manifestation (Peng, 2004). Emotions have an important impact on people’s production and life, physical and mental health, and interpersonal relationships. For example, for patients with depression or schizophrenia, abnormal emotions are the main clinical manifestations. If negative emotions can be identified before the onset, medical staff can intervene and treat in time. For the field of human-computer interaction, computer recognition can be realized, understand and adapt to human emotions, the human-computer interaction environment is more natural (Nie et al., 2012). Therefore, the decoding and recognition of emotions is an important research goal in the field of emotion computing.

Common emotion recognition methods are mainly divided into two categories: recognition based on non-physiological signals and recognition based on physiological signals. Recognition based on non-physiological signals mainly includes expression recognition and speech recognition, but these two methods have the risk of artificial disguise. In contrast, physiological signals can objectively reflect the true emotional state of a person. Physiological signals caused by emotions include heart rate, respiration, skin temperature, electromyography, electroencephalogram (EEG), and so on. Among them, EEG is not easy to be disguised, and the recognition rate is higher than other physiological signal recognition methods, so it is increasingly used in emotion recognition research (Nie et al., 2012).

Brain-computer interface (BCI) directly connects the brain and external devices, and realizes the information exchange between the brain and the device by decoding EEG (Wolpaw et al., 2000). With the rapid development of BCI and emotional computing, emotional BCI (e-BCI) that automatically recognize emotions have received extensive attention from all walks of life (Fattouh et al., 2013). Among them, decoding the individual’s emotional state from EEG information is the core content and key technology of the e-BCI (Molina et al., 2009).

So far, there are many EEG-based emotion recognition methods, and wavelet transform is one of the widely used ones. For example (Asghar et al., 2020) used the wavelet transform method to represent the EEG as a two-dimensional time-frequency distribution image, and then used a neural network method based on deep feature clustering (DFC) to evaluate the emotional state of the subjects, and achieved the recognition accuracy of 81.3% for four types of emotional states. On the basis of wavelet transform (Zhou et al., 2020), extracted Mel-frequency cepstral coefficient (MFCC) features, fused EEG features, and used deep residual network (Resnet18) to recognize two kinds of emotions in wake-up and price effect dimensions, with recognition accuracy of 86.01 and 85.46%. Luo et al. (2020) studied three algorithms of discrete wavelet transform (DWT), variance and fast fourier transform (FFT) to extract features of EEG signals, and spike neural network (SNN) to further classify the emotion signal, the two types of recognition accuracy of valence, arousal, dominance, and liking dimensions are 74, 78, 80, and 86.27%, respectively. Mohammadpour et al. (2017) used DWT to extract features, and then used artificial neural networks (ANN) performs emotion classification and achieves a recognition accuracy of 55.58% for six types of emotional states. Wei et al. (2020) used dual tree-complex wavelet transform (DT-CWT) to decompose and reconstruct EEG, and then extract features from time domain, frequency domain and non-linear analysis and use different integration strategies to obtain the recognition accuracy of the three types of emotions is 83.13%.

Although the wavelet transform can perform positioning in the time domain and the frequency domain at the same time, it is very convenient to perform the round-trip transform between the time domain and the frequency domain for time-varying signals, but a single wavelet basis function of the wavelet transform is difficult to accurately represent the local characteristics of the signal. It is easy to lose the original time domain characteristics when reconstructing the signal. Therefore, a new tunable Q-factor wavelet transform (TQWT) has been proposed in recent years (Selesnick, 2011). Compared with traditional wavelet transform, TQWT is more flexible and can better reflect complex oscillation signals including EEG by adjusting parameters, so it has quickly attracted the attention of scholars in related fields.

However, the decomposition of the signal also increases the amount of data to identify the features, which affects the performance of the system. This study introduces a feature selection algorithm to solve this problem. Traditional feature selection methods include principal component analysis (PCA), least absolute shrinkage and selection operator (LASSO), and recursive feature elimination (RFE), etc. (Mao et al., 2007). Among them, the binary gray wolf optimization (BGWO; Too et al., 2018) is an improved version of the gray wolf optimization (GWO; Sm et al., 2014), which was also inspired by the prey hunting activities of the gray wolf. An optimized search method of, it has the characteristics of strong convergence performance, few parameters, and easy implementation. It has been used in many fields by many researchers.

Therefore, this manuscript proposes a joint EEG recognition algorithm based on TQWT and BGWO. The algorithm first decomposes the sub-band from the original emotional EEG, and then extracts the signal sample entropy, Hjorth parameter, second-order difference mean and normalized second-order difference mean as features, and then optimizes the feature set through BGWO, and finally input to support vector machine (SVM) for classification. The follow-up structure of this article is as follows: First, the experimental materials and methods are described, including; the relevant description of the experimental data, the basic process of the TQWT algorithm, the feature extraction index, the basic process of the BGWO algorithm, and the classifier and algorithm evaluation index. The result part shows the classification effect of the algorithm on the data set, the analysis of the influence of different decomposition sub-bands and experimental parameters on the experimental results, and the comparative analysis with the classification effect of the classic algorithm. Finally, the experimental results are summarized and discussed.

## Materials and Methods

### Experimental Data and Preprocessing

**FIGURE 1:**
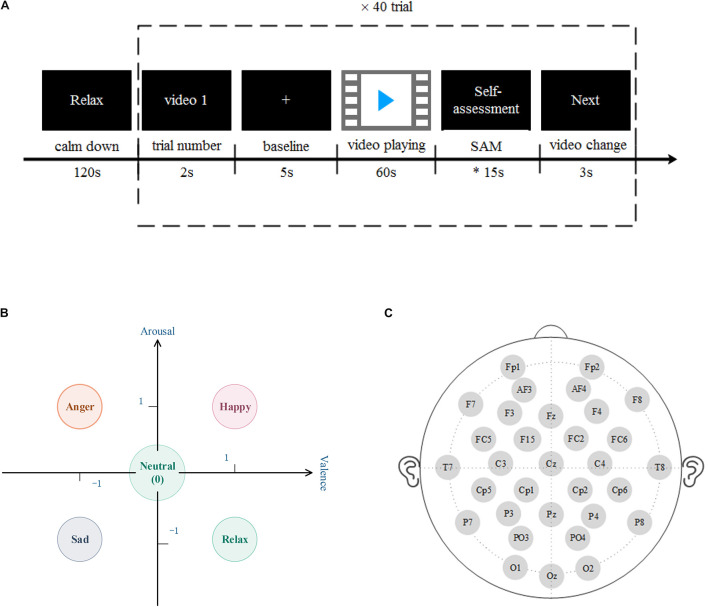
**(A)** The experimental paradigm used in the database for emotion analysis using physiological signals (DEAP) dataset. Before each subject’s experiment, the resting state signal was collected for 2 min; the trial serial number of 2 s was displayed to remind the subject of the current experimental progress; the baseline acquisition was conducted for 5 s, which represented the beginning of the recording of electroencephalogram (EEG); the MV was played for 1 min, and then the subject It takes about 15 s to fill in the SAM scale, and then after 3 s video conversion time, repeat the trial 40 times. **(B)** The five types of emotion models in this study. Including neutral, happy, anger, sad, and relax. **(C)** DEAP collects EEG according to the 32 leads selected by the international 10–20 system, which are Fp1, AF3, F3, F7, FC5, FC1, C3, T7, CP5, CP1, P3, P7, PO3, O1, Oz, Pz, Fp2, AF4, Fz, F4, F8, FC6, FC2, Cz, C4, T8, Cp6, Cp2, P4, P8, PO4, and O2.

This research uses a database for emotion analysis using physiological signals (DEAP; Koelstra, 2012), and its experimental paradigm is shown in Figure 1A. The DEAP data set includes the multi-modal physiological signals induced by 32 subjects watching 40–60-s music video materials and the subjects’ ratings of the video’s valence, arousal, dominance, and liking. Among them, the physiological signals include: 32 channels of EEG, eight channels of peripheral physiological signals: ➀ current skin response, ➁ skin temperature, ➂ blood volume pulse, ➃ respiration, EMG ➄ main muscles and ➅ trapezius, ➆ horizontal, and ➇ vertical electrooculograms (EOGs). In terms of subjective evaluation, the experiment used self-assessment manikin (SAM; Morris, 1995) with a scale of 1–9 to quantify the participants’ ratings of the value, arousal, advantage, and liking of video-induced emotions.

In this study, we set the threshold to 3, and divide each emotion sample into three levels according to the 9 scales of valence and arousal, 1–3, 4–6, and 7–9, respectively, mapped to “−1,” “0,” and “1” on the rectangular coordinate system, five types of emotion recognition are performed in two dimensions (as shown in Figure 1B; Fang et al., 2021), each type. The emotion setting rules are as follows:

• Happy (label 1): valence = 1 and arousal = 1
• Anger (label 2): valence = 1 and arousal = −1
• Sad (label 3): valence = −1 and arousal = −1
• Relax (label 4): valence = −1 and arousal = 1
• Neutral (label 5): valence = 0 or arousal = 0

In this study, a 32-channel EEG in the data set was selected for emotion recognition. The position of the EEG channel is shown in Figure 1C. Downsample the EEG data to 128 HZ, remove the EOGs artifacts, filter the signal to 4–45 HZ through a band-pass filter, and perform a whole-brain average reference. Each piece of data includes 60 s video-induced EEG data and 3 s video conversion.

The shape of the preprocessed EEG data of the 32 subjects is trial × channel × data, which is 40 × 32 × 8,064; the shape of the label data is trial × label (1–5), which is 40 × 1.

### Method

**FIGURE 2:**
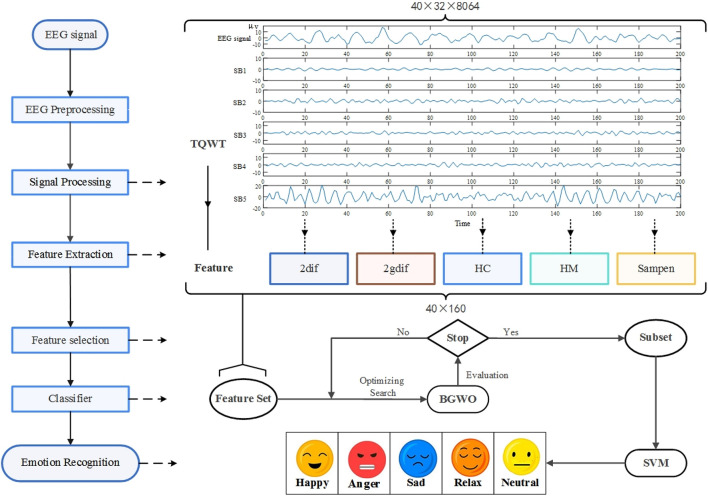
The method flow chart of this research. The research decomposes the pre-processed EEG input tunable-Q wavelet transform (TQWT) into multiple sub-bands (SB), extracts time-domain and non-linear features from the sub-bands, used binary gray wolf optimization (BGWO) to reduce dimensionality, selects the optimized subset as the classifier input, and finally passes support vector machine (SVM) Identify five types of emotions.

The algorithm flow is shown in Figure 2. In this study, the original EEG was preprocessed and decomposed into multiple sub-bands through TWQT, and then five features of sample entropy, second-order difference mean, normalized second-order difference mean, mobility and complexity were extracted from each sub-band., And then use BGWO to reduce the dimensionality of the feature set, and finally identify the five types of emotions: neutral, happy, anger, sad and relax through SVM.

#### Tunable-Q Wavelet Transform

Tunable-Q wavelet transform is a flexible DWT, a lifting algorithm based on wavelet transform, which can analyze complex oscillation signals more effectively (Selesnick, 2011), and has been used for the decomposition of EEG (Hassan et al., 2016). Its parameters are adjustable, so the transformation can be tuned and applied according to the oscillation behavior of the signal. The main parameters of TQWT are quality factor *Q*, total oversampling rate *r* and number of stages *J*. The degree to which *Q* affects the duration of wavelet oscillation is the ratio of its center frequency to its bandwidth. *r* is the total oversampling rate (redundancy) when calculating TQWT when *J* ≥ 1, that is, the total sampling rate coefficient of all sub-bands, which controls the excessive ringing of the system by affecting the scaling factor (*l*, *h;* Krishna et al., 2019). *J* represents the number of stages of the wavelet transform, which consists of a sequence of two-channel filter banks, and the low-pass output of each filter bank is used as the input of the continuous filter bank. The sub-bands (*J* + 1) obtained by signal decomposition are composed of the output signal of the high-pass filter of each filter bank and the output signal of the low-pass filter of the final filter bank (Selesnick, 2011).

The low-pass filter frequency response $H_{0}^{J}\,\left(\omega \right)$ and the high-pass filter frequency response $H_{1}^{J}\,\left(\omega \right)$ after level *J* should be defined as:

(1) $$H_{0}^{J}\,\left(\omega \right)=\left\{ \begin{array}{c} \prod_{m=0}^{J-1}H_{0}\,\left(\frac{\omega }{l^{m}}\right),\left|\omega \right|\leq l^{J}\,\pi \\ 0,l^{\text{J}}\,\pi <\left|\omega \right|\leq \pi \end{array}\right.$$

(2) $$H_{1}^{J}\,\left(\omega \right)=\left\{ \begin{array}{c} H_{1}\,\left(\frac{\omega }{l^{J-1}}\right)\,\prod_{m=0}^{J-2}H_{0}\,\left(\frac{\omega }{l^{m}}\right),\left(1-h\right)\,l^{J-1}\,\pi \leq \left|\omega \right|\leq l^{j-1}\,\pi \\ 0,\omega \in \left[-\pi ,\pi \right] \end{array}\right.$$

where low-pass scaling factor (*l*) and the high-pass scaling factor (*h*) are defined as:

(3) $$l=1-\frac{h}{r}$$

(4) $$h=\frac{2}{Q+1}$$

**FIGURE 3:**
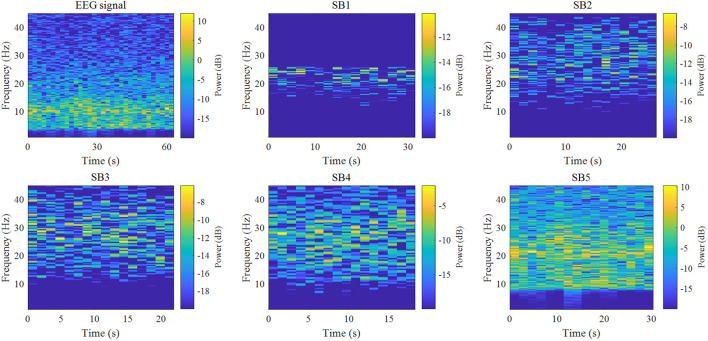
Time-frequency diagrams of five sub-bands obtained from single-channel EEG and TQWT decomposed signal. TQWT decomposes the EEG data of a single channel into five sub-bands (*Q* = 3, *r* = 3, and *J* = 4), among which sub-band 1 has energy fluctuations at 15–25 HZ, and both sub-bands 2 and 3 are at 10–45 HZ There are energy fluctuations from time to time. Sub-band 4 has energy fluctuations in the time-frequency axis within 4–45 HZ just like the original data, and the energy fluctuation of sub-band 5 is 8–45 HZ.

In this study, the EEG is decomposed into five sub-bands with a *Q* factor of 3 and an oversampling rate (*r*) of 3 by TQWT, and feature extraction from the sub-bands (*Q* = 3, *r* = 3, and *J* = 4). Figure 3 is a time-frequency diagram of TQWT decomposing the Fp1 channel EEG into five sub-bands.

#### Feature Extraction

Extract five time-domain non-linear features for each sub-band signal decomposed by TQWT, namely sample entropy, two differential features and two Hjorth parameters as classification features:

(1) Sample entropy

Sample Entropy (SampEn; Richman and Moorman, 2000) measures the complexity of time series by measuring the probability of generating a new pattern in the signal. It is similar to approximate entropy (AE) but is more consistent. Define the sample entropy of a finite array as:

(5) $$S\,a\,m\,p\,E\,n=-l\,n\,\left[A^{m}\,\left(r\right)/B^{m}\,\left(r\right)\right]$$

where ln represents the natural logarithm; *B^m^*(*u*) is defined as:

(6) $$B^{m}\,\left(u\right)=\frac{1}{N-m}\,\sum_{i=1}^{N-m}B_{i}^{m}\,\left(u\right)$$

(7) $$B_{i}^{m}\,\left(u\right)=\frac{1}{N-m-1}\,B_{i}$$

_*A^m^ u*_ is defined as:

(8) $$A^{m}\,\left(u\right)=\frac{1}{N-m}\,\sum_{i=1}^{N-m}A_{i}^{m}\,\left(u\right)$$

(9) $$A_{i}^{m}\,\left(u\right)=\frac{1}{N-m-1}\,A_{i}$$

where *m* represents the dimension of the vector, generally 1 or 2; *N* represents the length of the sequence; *u* represents the measure of “similarity,” generally choose *u* = 0.1 × *std*–0.25 × *std*, where *std* represents the standard deviation of the original time series. In this study, *m* = 2, *u* = 0.2 × *std*.

(2) Second-order difference mean (2dif)

(10) $$2\,\text{dif}=\frac{1}{N-2}\,\sum_{n=1}^{N-2}\left(x\,\left(n+2\right)-x\,\left(n\right)\right)$$

where *x*(*n*) represents the time series vector.

(3) Normalized second-order difference mean (2ndif)

(11) $$2\,\text{ndif}=\frac{2\,\text{dif}}{\sigma_{x}}$$

where _σ _x_ is the standard deviation.

(4) Hjorth parameter: mobility

The Hjorth parameter was proposed by Hjorth (1970). Among them, Hjorth-Mobility (HM) is a parameter to estimate the mean frequency, which measures the mobility of EEG:

(12) $$\text{HM}=\sqrt{\frac{v\,a\,r\,\left(\frac{\text{d}\,x\,\left(n\right)}{\text{d}\,n}\right)}{v\,a\,r\,\left(x\,\left(n\right)\right)}}$$

where var represents the variance.

(5) Hjorth parameter: complexity

Hjorth-Complexity (HC) is often used to estimate the bandwidth of the signal and measure the complexity of the EEG:

(13) $$\text{HC}=\frac{\text{Mobility}\,\left(\frac{\text{d}\,x\,\left(n\right)}{\text{d}\,n}\right)}{\text{Mobility}\,\left(x\,\left(n\right)\right)}$$

In this study, five types of features are extracted from the five sub-bands decomposed by TQWT, and the data shape of each sub-band is a trial feature, namely 40 × 32. A total of 32 feature matrices of 40 × 160 are obtained for subsequent feature selection.

#### Feature Selection

The feature matrix extracted from the TQWT sub-band is selected by the binary gray wolf optimization algorithm (BGWO; Too et al., 2018). The GWO algorithm is an optimized search method developed by simulating the hierarchy and hunting process of the wolf pack. The α, β, δ, and ω wolves in the wolf pack represent different social classes, respectively. This algorithm has been used by many researchers in the research fields of feature selection, parameter optimization and motor control because of its considerable optimization performance and simplicity and ease of implementation (Wei et al., 2017).

Emary et al. (2016) proposes two BGWO algorithms (BGWO1 and BGWO2) are proposed for feature selection. Among them, BGWO1 uses a crossover operator to update the wolf’s position, while BGWO2 uses a crossover operator to update the wolf’s position, while BGWO2 updates the wolf by converting the position into a binary vectors position. In this study, the BGWO2 method will be selected to optimize the feature set by dimensionality reduction, and the formula is as follows:

(14) $$Y^{n}\,\left(t+1\right)=\left\{ \begin{array}{c} 1,\mathrm{if}\,S\,\left(\frac{{\text{Y}}_{1}^{\text{n}}+{\text{Y}}_{2}^{\text{n}}+{\text{Y}}_{3}^{\text{n}}}{3}\right)\geq r_{0}\\ 0,\mathrm{else} \end{array}\right.$$

where *r*_0_ is a random number in [0,1]; *t* is the number of iterations; *n* is the dimension of the search space; *Y*_1_, *Y*_2_, and *Y*_3_ are defined as binary steps affected by α, β, and δ wolves, respectively; *Y^n^*(*t* + 1) is iteration the updated binary position in dimension *n* at time *t*. *S*(*a*) is defined as:

(15) $$S\,\left(a\right)=\frac{1}{1+{\text{e}}^{\left(-10\,\left(a-0.5\right)\right)}}$$

This article discusses the optimization effect of BGWO in three situations: (1) Fusion of the sub-band data of 32 subjects, and optimization of the five feature sets through BGWO, and the data is reduced from 40 × 160 to 40 × 57–92; (2) Fusion All the test data are optimized for the feature sets of the five sub-bands, and the data is reduced from 1,280 × 32 to 1,280 × 7–17; (3) Fusion of all test and sub-band data, the optimized data length is reduced from 1,280,160 to 1,280 × 43–67 not waiting.

#### Classifier and Evaluation Index

This research uses a SVM classifier. The basic idea of SVM is to solve the separation hyperplane that can correctly divide the training data set and have the largest geometric interval (Hsu and Lin, 2002). Originally to solve the two-classification problem, it is now widely used in the recognition of multiple types of emotional EEG (Kawintiranon et al., 2016; Samara et al., 2017).

In order to evaluate the effectiveness of the method proposed in this manuscript, four indicators of accuracy (Acc), sensitivity (Sen), specificity (Spe) and Kappa coefficient (Chu et al., 2021) are calculated through 6-fold cross-validation. The calculation formula of each indicator is as follows:

(16) $$\text{Acc}=\frac{\text{TP}+\text{TN}}{\text{TP}+\text{TN}+\text{FP}+\text{FN}}\times 100\%$$

where TP refers to true positive, TN is true negative, FP is false positive, and FN is false negative.

(17) $$\text{Sen}=\frac{\text{TP}}{\text{TP}+\text{FN}}\times 100\%$$

(18) $$\text{Spe}=\frac{\text{TN}}{\text{FP}+\text{TN}}\times 100\%$$

(19) $$\text{Kappa}=\frac{\text{Acc}-p_{e}}{1-p_{e}}$$

where *p*_*e*_ is the completely random classification accuracy. For the five classification problems in this manuscript, *p_*e*_* = 0.2.

Accuracy is our most common evaluation index. Generally speaking, the higher the accuracy, the better the classifier. Sensitivity represents the proportion of all positive examples that are matched and measures the classifier’s ability to recognize positive examples. Specificity represents the proportion of all negative cases that are matched and measures the ability of the classifier to recognize negative cases. The Kappa coefficient is usually used for consistency testing. It can be used as an index to measure the accuracy of classification, and it can also be used as a normalized index to measure the accuracy of different classification numbers.

### Experimental Results

The experiment is carried out on MATLAB R2019b platform under Windows 8.1 64 bit operating system. The system CPU is AMD Radeon R5 and the memory is 8 GB. This study uses the DEAP data set to verify the effectiveness of the algorithm for emotion recognition from five aspects: (1) The data of each subject is decomposed by TQWT, and the features are extracted after fusing the sub-bands. The feature sets are classified by SVM before and after BGWO. In order to explore the classification performance of the algorithm to individuals, and the improvement effect of BGWO on the algorithm. (2) Extract features from each sub-band decomposed by TQWT, and merge the feature sets of all subjects into SVM classification before and after BGWO to explore the classification performance of the algorithm for different sub-bands of TQWT. (3) Fuse the data of the subject and the sub-bands, and the total feature set obtained after BGWO optimization is used as the classification feature to explore the overall recognition performance of the algorithm. (4) On the basis of experiment (1), the influence of key parameters of the algorithm on the accuracy of individual recognition is explored. (5) Compare and analyze other EEG emotion recognition methods of the same data set, in order to explore the effectiveness of this method for multi-type emotion recognition.

### Accuracy of Individual Recognition

**FIGURE 4:**
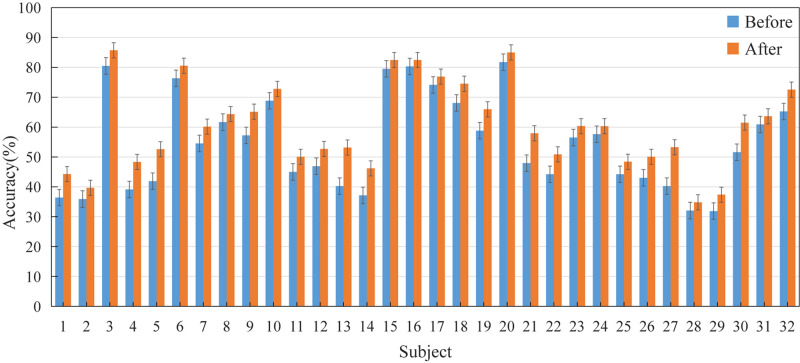
The accuracy information of the feature set before and after BGWO. Where before refers to the average recognition accuracy ± standard error of the five types of feature sets without BGWO optimization; After refers to the average recognition accuracy ± standard error of the feature set after BGWO optimization.

The EEG of 32 subjects were decomposed into five sub-bands by TQWT, the sub-bands were fused and features were extracted and then classified by SVM, the emotion recognition accuracy rate of each subject was obtained as shown in Figure 4. Among them, “Before” indicates that the feature set is not optimized by BGWO, and “After” indicates the accuracy information after feature selection by BGWO. Judging from the recognition accuracy of the five categories in the figure, the average recognition accuracy of the two differences, two Hjorth parameters and sample entropy as the classification features is 53.37%; the maximum recognition accuracy of the individual is 87.7%, appearing in 20th subject. After the feature set is optimized by BGWO, the average recognition accuracy of the five types of features is 60.44%; the maximum recognition accuracy of the individual is 88.1%, which appears in the 18th subject. The accuracy of each participant increased by 7.07% on average.

In order to show the time complexity of the algorithm, Table 1 counts the time consumption information of the 63 s emotion recognition process of a single trial.

**TABLE 1:** Time consumption statistics of this research method(s).

**Subject**	**TQWT**	**Feature extraction**	**BGWO**	**SVM**	**Total time**
1	0.0052	0.0797	1.0827	0.7377	1.9850
2	0.0046	0.0789	1.1811	0.5733	1.9168
3	0.0055	0.0830	1.1099	0.2136	1.4950
4	0.0049	0.0815	1.1091	0.4496	1.7266
5	0.0054	0.0812	1.1513	0.5508	1.8699
6	0.0055	0.0820	1.1019	0.2911	1.5625
7	0.0047	0.0825	1.0904	0.3029	1.5630
8	0.0053	0.0808	1.1171	0.5324	1.8164
9	0.0048	0.0848	1.1645	0.3213	1.6602
10	0.0045	0.0800	1.1011	0.5356	1.8012
11	0.0047	0.0822	1.1166	0.6990	1.9847
12	0.0059	0.0796	1.1249	0.3097	1.5997
13	0.0048	0.0791	1.1123	0.4574	1.7327
14	0.0045	0.0807	1.1144	0.6253	1.9056
15	0.0052	0.0825	1.1876	0.4849	1.8427
16	0.0046	0.0801	1.1382	0.4296	1.7326
17	0.0063	0.0829	1.1028	0.2028	1.4777
18	0.0062	0.0792	1.2041	0.1932	1.5619
19	0.0050	0.0803	1.3384	0.5900	2.0940
20	0.0060	0.0787	1.4798	0.2691	1.9123
21	0.0050	0.0845	1.1740	0.2876	1.6356
22	0.0047	0.0812	1.2576	0.4934	1.9181
23	0.0059	0.0804	1.3163	0.4459	1.9289
24	0.0051	0.0810	1.2210	0.2974	1.6855
25	0.0056	0.0869	1.1947	0.2966	1.6707
26	0.0054	0.0884	1.2080	0.5649	1.9551
27	0.0044	0.0802	1.3141	0.3100	1.7889
28	0.0056	0.0814	1.1724	0.6294	1.9702
29	0.0054	0.0838	1.2144	0.6628	2.0502
30	0.0048	0.0828	1.2136	0.3896	1.7736
31	0.0056	0.0803	1.2812	0.4850	1.9324
32	0.0048	0.0820	1.1622	0.2938	1.6248

### The Recognition Accuracy of Different TQWT Sub-Bands

The feature sets of 32 subjects were fused, and the feature matrixes of five sub-bands were respectively, passed through BGWO, and the classification accuracy before and after optimization of each feature was obtained as shown in Table 2. It can be seen from the table that the classification accuracy of each feature when the five sub-bands are not optimized are 57.168 ± 1.34, 58.36 ± 2.08, 58.28 ± 1.34, 58.28 ± 1.22, and 57.578 ± 1.34%, respectively. The recognition accuracy of each sub-band after BGWO was 63.36 ± 2.64, 62.89 ± 1.32, 62.764 ± 2.07, 62.768 ± 0.95, and 62.734 ± 1.88%, and the Acc of each sub-band increased by 4.97 ± 0.28% on average.

**TABLE 2:** Statistics of accuracy (%) of various features of each sub-band before and after binary gray wolf optimization (BGWO).

**BGWO**	**Feature**	**Sub-band**				
		**SB1**	**SB2**	**SB3**	**SB4**	**SB5**
Before	2dif	57.81	58.2	58.59	57.81	**59.38**
	2ndif	58.98	58.59	60.16	57.42	56.64
	HC	55.86	61.33	58.59	59.37	58.59
	HM	55.86	55.47	56.64	57.03	56.25
	Sampen	57.42	58.21	57.42	59.77	57.03
	Average	57.186	58.36	58.28	58.28	57.578
	Std	1.34	2.08	1.34	1.22	1.34
After	2dif	63.67	63.28	61.72	61.36	62.11
	2ndif	**67.58**	**64.45**	**66.41**	63.28	62.11
	HC	60.94	63.67	61.33	**63.67**	**65.62**
	HM	61.33	61.72	62.25	62.25	63.28
	Sampen	63.28	61.33	62.11	63.28	60.55
	Average	63.36	62.89	62.764	62.768	62.734
	Std	2.64	1.32	2.07	0.95	1.88

*Bold values means the maximum accuracy of each sub-band.*

### Recognition Results of Fusion of Subjects and Sub-Band Data

Table 3 counts the accuracy (All Acc)%, sensitivity (Sen)%, specificity (Spe)%, and Kappa coefficient information of the five types of emotion recognition overall data (fusion of subjects and sub-band data) with five types of features. The overall recognition accuracy of the algorithm in this manuscript is 62.34%, the average sensitivity is 65.22%, the average specificity is 78.13%, and the Kappa coefficient is 0.53. It can be seen that the classification performance of time-domain non-linear features is similar in accuracy and Kappa coefficient; the optimal performance of sensitivity and specificity are both differential features.

**TABLE 3:** The overall accuracy (All Acc)%, sensitivity (Sen)%, specificity (Spe)%, and Kappa coefficient of five types of emotion recognition based on tunable-Q wavelet transform (TQWT) and BGWO.

	**All Acc**	**Sen**	**Spe**	**Kappa**
2dif	62.1	**70.25**	81.33	0.5263
2ndif	62.5	64.19	**82.01**	0.5313
HC	**62.6**	62.12	79.29	**0.5325**
HM	62.4	65.13	68.7	0.53
Sampen	62.1	64.4	79.3	0.5263
Average	62.34	65.22	78.13	0.53
Std	0.23	3.03	5.41	0.003

*All means fusion of all subjects and sub-band data. Bold value means the optimal value of each indicator.*

### The Impact of Key Parameters on Recognition Accuracy

Tunable-Q wavelet transform can adjust three parameters to apply to different individuals and achieve the best classification effect. Selesnick (2011) provides suggestions for the selection of TQWT parameters, that is, *Q* ≥ 1, *r* value of 3.0 or 4.0, and *J* ≥ 1. On this basis, this article will specifically explore the influence of parameters on the accuracy of different individual emotion recognition. Through repeated trials, the optimal recognition accuracy of 32 subjects and their corresponding parameter combinations are shown in Table 4. It can be seen from the table that the average recognition accuracy rate of the subjects obtained after the personalized parameters is 65.2%, and the accuracy rate increases to 68.24% after passing BGWO.

**TABLE 4:** The optimal recognition accuracy of different individuals and their corresponding TQWT parameter combinations.

**Subject**	**TQWT parameter**			**Accuracy (%)**	
	***Q***	***r***	***J***	**Before BGWO**	**After BGWO**
1	1	3	4	52.38	60.32
2	3	3	2	42.86	46.03
3	5	3	3	**89.68**	**90.48**
4	1	3	4	52.78	54.76
5	1	3	4	59.52	59.92
6	1	3	2	82.14	83.33
7	1	3	1	59.52	63.1
8	4	3	1	67.86	74.21
9	1	3	4	77.38	77.78
10	3	3	1	79.76	80.56
11	1	3	3	57.94	58.33
12	4	3	3	57.54	58.73
13	4	3	1	52.38	55.95
14	1	3	5	57.54	59.92
15	2	3	3	85.71	87.3
16	5	3	3	85.32	85.71
17	3	3	3	82.94	83.33
18	3	3	1	70.24	73.41
19	2	3	6	68.25	69.84
20	3	3	1	88.10	88.10
21	4	3	4	62.70	72.22
22	4	3	1	51.59	55.16
23	1	3	3	67.86	72.22
24	1	3	3	70.24	75.40
25	5	3	6	54.37	61.9
26	1	3	6	51.98	60.32
27	1	3	1	55.16	57.14
28	3	3	1	42.46	43.25
29	1	3	4	45.63	48.41
30	1	3	1	67.06	75.4
31	3	3	1	73.02	73.41
32	3	3	3	74.60	77.78
Average				65.20	68.24
Std				13.72	12.99

*Bold values indicate the subject maximum accuracy.*

**FIGURE 5:**
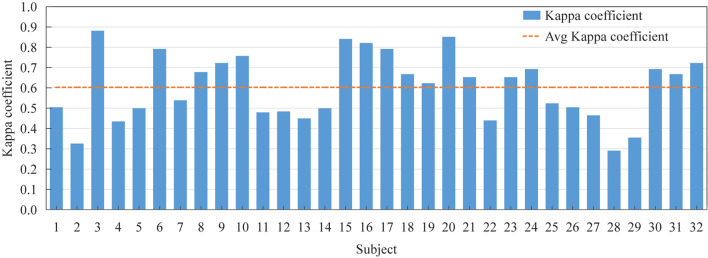
The Kappa coefficient of the optimal parameter combination of each subject and the average Kappa coefficient of all subjects.

Figure 5 shows the Kappa coefficient of the best combination of parameters for each subject. It can be seen from the figure that the maximum Kappa coefficient is 0.88, and the average Kappa coefficient is 0.603. The Kappa coefficients of different subjects are quite different.

### Comparative Analysis of Related Research

Table 5 compares some EEG emotion recognition research methods based on the DEAP data set, and normalizes the classification accuracy of different categories into the Kappa coefficient. Among them, the creator of this database (Koelstra, 2012) used the correlation coefficient to do a 2-classification study, and the Kappa coefficients of the arousal, valence and dominance dimensions were 0.24, 0.15, and 0.11. Yin et al. (2020) used locally-robust feature selection (LRFS) method is used to conduct a 2-classification study, and the Kappa coefficients of the arousal and valence dimensions are 0.3 and 0.36. Gupta et al. (2016) used the graph-theoretic feature extraction method for three classification studies, the Kappa coefficients in four dimensions were 0.54, 0.51, 0.48, and 0.48. Tao and Dan (2021) proposed a multi-source co-adaptation framework for mining diverse correlation information (MACI), the Kappa coefficient of the three categories was 0.45. Zhang et al. (2016) used the ReliefF feature selection method to conduct the four-category study, and the Kappa coefficient of the category was 0.45. Gupta et al. (2019) used flexible analytic wavelet transform (FAWT) Extract features for four classification studies, the Kappa coefficient was 0.45. Atkinson and Campos (2016) proposed an emotion recognition model combining the feature selection method based on minimum-Redundancy-Maximum-Relevance (mRMR) and kernel classifier. The Kappa coefficients of the two categories were 0.46 and 0.46, the kappa coefficients of the three categories were 0.43 and 0.41, and the Kappa coefficients of the five categories were 0.33 and 0.32, respectively. Generally speaking, the higher the number of machine learning classifications, the lower the classification accuracy (Kong et al., 2021). It can be seen from the table that the classification performance of this method has reached a 5-class Kappa coefficient of 0.603. Compared with the classification performance of the above methods, this research has achieved outstanding classification effects.

**TABLE 5:** Comparison of classification performance between the method in this manuscript and other studies on the database for emotion analysis using physiological signals (DEAP) dataset.

**Authors**	**Method**	**Accuracy (%)**				**Kappa**
		**2 Classes**	**3 Classes**	**4 Classes**	**5 Classes**	
Koelstra, 2012	Correlation coefficient + NB	62, 57.6, 55.4	–	–	–	0.24, 0.15, 0.11
Yin et al., 2020	LRFS + LSSVM/NB	65,68	–	–	–	0.30, 0.36
Gupta et al., 2016	Graph-theoretic + SVM/RVM	–	69, 67, 65, 65	–	–	0.54, 0.51, 0.48, 0.48
Tao and Dan, 2021	MACI	–	63.31	–	–	0.45
Zhang et al., 2016	ReliefF + SVM	–	–	58.75	–	0.45
Gupta et al., 2019	FAWT + SVM	–	–	59.06	–	0.45
Atkinson and Campos, 2016	mRMR + SVM	73.14, 73.06	62.33, 60.7	–	46.69, 45.32	0.46, 0.46/0.43, 0.41/0.33, 0.32
This work	TQWT-BGWO + SVM	–	–	–	68.24	0.603

## Discussion

In this study, for the EEG-based e-BCI, the method of TQWT and BGWO was used to identify the five types of emotions in the DEAP dataset: neutral, happy, sad, relax, and anger. First of all, in terms of the number of identifications, this research has improved compared with previous traditional studies, and increased the types of signal identification. Secondly, for EEG-based emotion recognition, the current more innovative TQWT algorithm is selected to analyze the signal. In addition, for the EEG feature selection method, BGWO is used for the first time to optimize the EEG emotional features, and its optimization effect on the emotion recognition task is verified.

It can be seen from Figure 4 that it is feasible to extract the time domain and non-linear dynamic characteristics from TQWT and use SVM to identify five types of emotions. The classification accuracy of different subjects for the same trial is significantly different, indicating that the same emotion-inducing material has different emotion-inducing effects for different subjects. Excluding subjects or trials with poor emotion-inducing effects may improve the overall recognition accuracy. Therefore, designing an emotion-induced paradigm suitable for different subjects is still a prominent problem of e-BCI. Secondly, Figure 4 shows that after using BGWO, the accuracy of a single subject has been enhanced, showing better applicability. In addition, it can be seen from part Feature Selection that BGWO can effectively reduce the data size of the feature set. It shows that BGWO is also an effective optimization method for EEG emotion recognition tasks. It can be seen from Table 1 that the time consumption of each stage of the method proposed in this manuscript basically meets the online BCI system.

It can be seen from Table 2 that the optimal classification accuracy can be obtained by taking the TQWT sub-band as the classification axis. In addition, the standard deviation *Std* ≤ 2.64 of the classification accuracy of the same classification feature in different sub-bands indicates that the stability of the recognition accuracy of the TQWT decomposition signal is good. Table 3 shows the accuracy, sensitivity, specificity, and Kappa coefficient of the five features under this research method. Among them, the average accuracy rate is 62.34%, which exceeds the probability of random guessing (above-chance level) by 42.34%. For sensitivity and specificity, this method has a 65.22% ability to recognize positive cases and 78.13% on negative cases. If understood from a medical point of view, sensitivity and specificity measure the missed diagnosis rate and the misdiagnosis rate, respectively. The Kappa coefficient is 0.53, which represents the ratio of the error reduction of the classification and the chance level.

Table 4 statistics the optimal TQWT parameter combination of each subject and the recognition accuracy information before and after the obtained BGWO. Compared with Figure 4, tuning the TQWT parameters for different subjects can achieve better recognition performance, achieving a maximum individual recognition accuracy of 90.48% and an average recognition accuracy of 68.24%. Figure 5 shows the Kappa coefficient information of the subjects. It can be seen that the average Kappa coefficient of all subjects is 0.603, and the Kappa coefficients of all subjects are linearly related. In addition, for all subjects, the optimal value of *Q* factor is between 1 and 5, the optimal value of *r* is both 3, and the optimal value of *J* is between 1 and 6. Individual differences are not only manifested in the inducing effect of emotions, but also in system parameters. Therefore, TQWT with adjustable parameters is an effective method to overcome individual differences. It is worth noting that the EEG emotion recognition methods based on TQWT and BGWO use simple and common features and classifiers. If try other advanced or improved features and classifiers, can achieve good classification results, or you can switch the emotion category It is a control instruction for BCI equipment, which will be more conducive to the development of e-BCI.

## Conclusion

In this study, the TQWT-BGWO method was used to recognize five types of emotions from EEG. TQWT decomposes the EEG into sub-bands, extracts features from the sub-bands, and used the SVM classifier to classify after BGWO optimization to realize the recognition of five types of emotion signals: neutral, happy, sad, relaxed, and anger. The parameterized TQWT signal decomposition can overcome individual differences to a certain extent, and combined with the BGWO feature selection method with fast convergence speed and good optimization performance, it can effectively improve the recognition accuracy of the system. Through the DEAP data set, the effectiveness of the proposed algorithm is verified. The experimental results show that the research method in this manuscript has an average recognition accuracy of 68.24%, a sensitivity of 65.22%, a specificity of 78.13% and a Kappa coefficient of 0.603 for the five types of emotions. The proposed algorithm can effectively identify multiple types of emotional states, and provides new ideas for emotional BCIs.

## Data Availability Statement

The original contributions presented in the study are included in the article/supplementary material, further inquiries can be directed to the corresponding authors.

## Author Contributions

SL and XL conceived the experiment. SL carried out the experiment and data analysis. LZ, ZC, and AG provided the suggestions. AG and YF revised the manuscript. All authors contributed to the article and approved the submitted version.

## Conflict of Interest

The authors declare that the research was conducted in the absence of any commercial or financial relationships that could be construed as a potential conflict of interest.

## Publisher’s Note

All claims expressed in this article are solely those of the authors and do not necessarily represent those of their affiliated organizations, or those of the publisher, the editors and the reviewers. Any product that may be evaluated in this article, or claim that may be made by its manufacturer, is not guaranteed or endorsed by the publisher.
